# Global Perspective on Kidney Transplantation: Perú

**DOI:** 10.34067/KID.0000001209

**Published:** 2026-03-26

**Authors:** Natalia Nombera-Aznaran, Domingo Chang, Franco Cabeza Rivera

**Affiliations:** 1 Division of Nephrology, Department of Internal Medicine, University of Alabama at Birmingham, Birmingham, Alabama; 2 Division of Nephrology, Hospital Nacional Dos de Mayo, Lima, Peru; 3 Katz Family Division of Nephrology and Hypertension, University of Miami Miller School of Medicine, Miami, Florida

**Keywords:** kidney transplantation, renal transplantation, social determinants of health, transplantation

## Introduction

Perú, the third largest country in South America (1,285,216 km^2^; population 34.2 million), is characterized by marked geographic and demographic heterogeneity. Although the country has sustained economic growth over recent decades, it continues to face a significant burden of kidney disease. CKD is a growing public health concern, with an estimated prevalence of 16.8%.^1^ Between 2003 and 2015, CKD accounted for roughly 2.0% of recorded deaths; however, underdiagnosis and misclassification suggest the actual burden may be higher.^2^ Awareness remains limited: Nearly 90% of individuals with CKD are believed to be unaware of their condition,^3^ often resulting in delayed diagnoses at advanced disease stages.

In 2020, approximately 20,000 patients were receiving dialysis.^4^ However, fewer than 1% of individuals with kidney failure underwent transplantation, reflecting a substantial gap in access to definitive KRT.^4^

## National Transplant Activity

From 2010 to 2024, cumulative national registry data indicate more than 2200 kidney transplants were performed in Perú.^4^ Nevertheless, transplant rates per million population (pmp) remain among the lowest in South America. According to the Latin American registry of Dialysis and Renal Transplantation, Perú had 63.2 living patients pmp with functioning grafts and a transplant rate of 6.1 pmp, compared with regional averages of 156 patients pmp and 19.4 transplants pmp.^5^ These reported rates suggest persistent disparities in access relative to neighboring countries with similar dialysis prevalence. A limited number of transplant centers, underdeveloped referral networks, and logistical challenges in organ allocation contribute to these low rates.

## Pediatric Kidney Transplantation

Children account for roughly 11% of all kidney transplant recipients nationally.^4^ Regionally, Perú represented just 3% of pediatric transplants, ranking seventh among participating Latin American countries.^6^ Pediatric recipients often experience prolonged dialysis exposure before transplantation. The mean duration on dialysis before transplant is approximately 3.2 years, exceeding the 2.0-year average observed in adults.^6^ This difference may reflect delayed referral, limited pediatric transplant capacity, and restricted availability of both deceased and living donor organs.

## Donor Type and Pandemic Impact

Living donors accounted for slightly more than one-quarter of kidney transplants between 2010 and 2024.^4^ For most of this period, living donor rates remained below 1.5 pmp, with modest increases to 2.36 pmp in 2023 and 2.2 pmp in 2024.^4^

The coronavirus disease 2019 pandemic had a notable effect on transplant activity, particularly among living donors. Between 2019 and 2024, only a single unrelated living donor transplant was reported nationwide, reflecting both system strain and interruptions in elective surgical procedures.^4^ The pandemic also highlighted the vulnerability of transplant programs in resource-limited settings, demonstrating the need for contingency planning, prioritization protocols, and robust infection control measures to maintain continuity of care.

## Health Care System and Geographic Disparities

Perú’s public health care system is divided among EsSalud (covering formally employed workers and their dependents), Seguro Integral de Salud (SIS, primarily covering low-income populations), and the health systems of the armed forces and police. Historically, EsSalud performed nearly 90% of solid organ transplants.^4^

Registry data from 2019 to 2024 indicate that SIS accounted for only 12.1% of kidney transplants, with other institutions contributing <2%.^4^ This distribution highlights the role of insurance affiliation in determining access and suggests inequities on the basis of employment status and socioeconomic factors.

**Figure 1:**
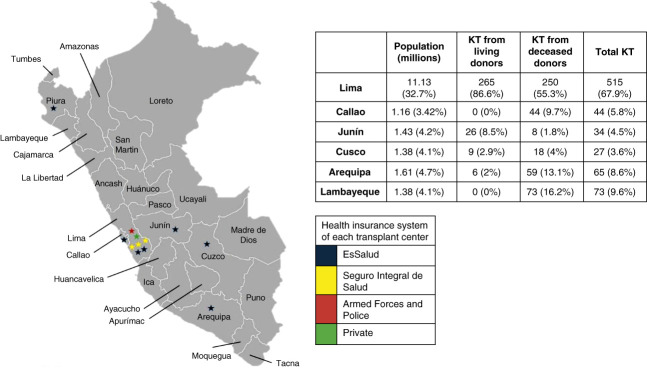
**Geographic distribution of KT centers and KT volume by region in Perú, 2019–2024.** The map shows Peru's administrative regions (including Callao) and the locations of kidney transplant centers (stars). Star color indicates the health insurance system of each transplant center: EsSalud (blue), Seguro Integral de Salud (yellow), Armed Forces and Police (red), and private (green). All regions are labeled. The accompanying table summarizes the population (millions) and the number of KT from living donors, deceased donors, and total KTs for the regions shown (percentages in parentheses). *Base map*: Simplemaps (Perú Admin-1 SVG), used under a free license for personal and commercial use. KT, kidney transplant; SIS, Seguro Integral de Salud; SVG, scalable vector graphics.

Given that geographic centralization is pronounced, transplantation services are available in only five of Perú’s 25 regions. Lima accounted for 73.8% of procedures, followed by Lambayeque (9.6%), Arequipa (8.6%), Junín (4.49%), and Cusco (3.6%).^4^ (Figure 1). While Lima comprises roughly 30% of the population, this concentration creates considerable barriers for patients living residing elsewhere.

## Legal Framework and Allocation

Organ donation and transplantation in Perú are regulated by Law No. 28189 (2004) and Law No. 30473 (2016), which established the National Transplant Organization and defined the legal basis for deceased and living donation.^7^ Despite this regulatory framework, allocation remains operationally fragmented. Transplant programs were historically developed within individual insurance networks, rather than as part of a unified national system. Consequently, deceased-donor kidneys are largely distributed within the insurance subsystem to which the donor belongs, resulting in parallel waiting lists rather than a single, unified national registry. This structure reflects the broader segmentation of the public health care system and effectively links organ distribution to insurance affiliation. Such fragmentation reduces the effective donor–recipient pool, limits matching efficiency, and may prolong waiting times, particularly for patients insured through SIS.

International analyses of global transplant capacity have shown that countries lacking centralized allocation systems tend to report lower transplant rates and greater inequities in access.^8^ Establishing a consolidated national waiting list in Perú, while maintaining financial responsibility within existing insurance schemes, would represent a feasible structural reform to enhance fairness, optimize organ utilization, and reduce disparities in access to transplantation.

## Economic Considerations

The estimated cost of kidney transplantation in Perú is approximately USD 45,000, intended to be fully covered by public insurance.^9^ This estimate includes surgical procedures, immunosuppressive therapy, routine follow-up, and potential hospital readmissions over the first 5 years. Although initial costs exceed those of dialysis, cumulative expenditure over 5 years is lower, supporting the long-term cost-effectiveness of transplantation.^9^

## Outcomes and Data Gaps

National data on long-term outcomes remain limited. Most evidence derives from single-center retrospective studies. A 27-year experience from a regional center reported delayed graft function in 14.8% of recipients and chronic graft dysfunction in 11.1%. Fifteen-year patient survival was approximately 60%, with superior outcomes among recipients of living donor kidneys.^10^

Follow-up of living donors is limited but encouraging. In one cohort with a mean follow-up of 5.14 years, 85.7% maintained estimated glomerular filtration rates above 60 ml/min per 1.73 m^2^, with no cases of ESKD or donor mortality.^10^

The establishment of a national kidney transplant registry is needed to systematically assess graft survival, patient outcomes, and donor safety; identify gaps in care; and inform evidence-based clinical and policy decisions.

## Sociocultural Barriers

Organ donation rates remain low. Qualitative studies have highlighted persistent misconceptions, such as beliefs that brain death is reversible, that recipients may acquire donor characteristics, or that religious doctrines forbid donation. These misunderstandings contribute to high family refusal rates and limit deceased donation.^10^ Community engagement, culturally sensitive educational campaigns, and targeted interventions involving religious and community leaders may increase consent rates and awareness.

## Conclusions

Despite more than two decades of kidney transplantation, Perú continues to report some of the lowest rates in South America. Key structural constraints include insurance fragmentation, geographic centralization, lack of a unified waiting list, and limited living donation. Expanding transplant capacity beyond Lima, integrating services across public insurance systems, establishing a national waiting list and outcomes registry, and implementing educational initiatives to address misconceptions are critical to improving equity and access nationwide. Policy reforms, infrastructure development, and coordinated public health strategies are necessary to support sustainable improvements in kidney transplant care.

## Supplementary Material
