# Supplementary material for: Global Perspective on Kidney Transplantation: Perú
Source: Kidney360. 2026 Mar 26;7(5):1144–6. doi: 10.34067/KID.0000001209 (PMC13229426; doi:10.34067/KID.0000001209)
Supplement: Supplementary file 1 [file kidney360-7-1144-s001.pdf]

ASN American Society of Nephrology | JASN Journal of the American Society of Nephrology | KIDNEY360 Accessing Our World From Every Angle | CJASN Clinical Journal of the American Society of Nephrology

ASN Journal Disclosure Form

As per ASN journal policy, I have disclosed any financial relationships or commitments I have held in the past 36 months as included below. I have listed my Current Employer below to indicate there is a relationship requiring disclosure. If no relationship exists, my Current Employer is not listed.

F. Cabeza Rivera has nothing to disclose.

I understand that the information above will be published within the journal article, if accepted, and that failure to comply and/or to accurately and completely report the potential financial conflicts of interest could lead to the following: 1) Prior to publication, article rejection, or 2) Post-publication, sanctions ranging from, but not limited to, issuing a correction, reporting the inaccurate information to the authors' institution, banning authors from submitting work to ASN journals for varying lengths of time, and/or retraction of the published work.

Name: Franco H. Cabeza Rivera
Manuscript ID: K360-2025-001544R1
Manuscript Title: Global Perspective on Kidney Transplantation: Perú
Date of Completion: March 17, 2026
Disclosure Updated Date: March 17, 2026

ASN American Society of Nephrology | JASN Journal of the American Society of Nephrology | KIDNEY360 Accessing Our World From Every Angle | CJASN Clinical Journal of the American Society of Nephrology

ASN Journal Disclosure Form

As per ASN journal policy, I have disclosed any financial relationships or commitments I have held in the past 36 months as included below. I have listed my Current Employer below to indicate there is a relationship requiring disclosure. If no relationship exists, my Current Employer is not listed.

D. Chang has nothing to disclose.

I understand that the information above will be published within the journal article, if accepted, and that failure to comply and/or to accurately and completely report the potential financial conflicts of interest could lead to the following: 1) Prior to publication, article rejection, or 2) Post-publication, sanctions ranging from, but not limited to, issuing a correction, reporting the inaccurate information to the authors' institution, banning authors from submitting work to ASN journals for varying lengths of time, and/or retraction of the published work.

Name: Domingo Chang
Manuscript ID: K360-2025-001544R1
Manuscript Title: Global Perspective on Kidney Transplantation: Perú
Date of Completion: March 17, 2026
Disclosure Updated Date: March 17, 2026

ASN
American Society of Nephrology
JASN
Journal of the American Society of Nephrology
KIDNEY360
Accessing Our World From Every Angle
CJASN
Clinical Journal of the American Society of Nephrology

ASN Journal Disclosure Form

As per ASN journal policy, I have disclosed any financial relationships or commitments I have held in the past 36 months as included below. I have listed my Current Employer below to indicate there is a relationship requiring disclosure. If no relationship exists, my Current Employer is not listed.

N. Nombera reports the following:
Employer: University of Alabama at Birmingham; and Research Funding: University of Alabama at Birmingham; Peruvian University Cayetano Heredia.

I understand that the information above will be published within the journal article, if accepted, and that failure to comply and/or to accurately and completely report the potential financial conflicts of interest could lead to the following: 1) Prior to publication, article rejection, or 2) Post-publication, sanctions ranging from, but not limited to, issuing a correction, reporting the inaccurate information to the authors' institution, banning authors from submitting work to ASN journals for varying lengths of time, and/or retraction of the published work.

Name: Natalia R. Nombera
Manuscript ID: K360-2025-001544R1
Manuscript Title: Global Perspective on Kidney Transplantation: Perú
Date of Completion: March 16, 2026
Disclosure Updated Date: March 16, 2026
